# Expression of LINE-1 retrotransposon in early human spontaneous abortion tissues

**DOI:** 10.1097/MD.0000000000031964

**Published:** 2022-12-09

**Authors:** Chao Lou, Rong Qiang, Hanzhi Wu, Liping Zhang, Wei Li, Ting Jia, Xing Liu

**Affiliations:** a Department of Genetics, Northwest Women’s and Children’s Hospital, Xi’an, Shaanxi Province, China; b Department of Obstetrics, Northwest Women’s and Children’s Hospital, Xi’an, Shaanxi Province, China.

**Keywords:** abortion, immunohistochemistry, induced, LINE-1 retrotransposon, RT-PCR, spontaneous, Western Blot

## Abstract

The aim of this study is to investigate a new mechanism that may affect spontaneous abortions (SA): Can long interspersed nuclear element-1 (LINE-1) insertions in embryo cells lead to early SA?

**Methods:**

The method involves prospective study on new mechanism of human early SA. Twenty SA tissues and 10 induced abortion (IA) tissues were utilized for this experiment. Western Blot, Immunohistochemistry (IHC), and reverse transcription-polymerase chain reaction were used to analyze different LINE-1 proteins and mRNA expression between early SA tissues and early IA tissues. SPSS software version 21.0 was used for statistical analysis.

**Results:**

Western Blot demonstrated that the LINE-1 protein expression in SA tissues (Mean: 60.2%) is higher than in IA tissues (Mean: 30.3%) in 91% of the compared samples. reverse transcription-polymerase chain reaction showed that LINE-1 mRNA expression in SA tissues (Mean: 64.2%) is higher than in IA tissues (Mean: 29.2%) in 6 primer pairs in 89% of the compared samples. IHC showed that the LINE-1 protein expression in SA tissues (Mean: 59.2%) is higher than in IA tissues (Mean: 28.8%) in 83% of the compared samples.

**Conclusions:**

Expression of LINE-1 in early SA tissues is higher than in IA tissues, LINE-1 may lead to early SA and LINE-1 plays a role in early SA, this shows that a new mechanism may be involved in SA.

## 1. Introduction

Long interspersed nuclear element-1 (LINE-1) in the human genome (LINE-1 or L1) is the only autonomously active retrotransposon, it has half a million copies and it accounts for 17% of the human genome.^[1–5]^ The full length of LINE-1 is 6000 bases, it contains 2 non-overlapping open reading frames (ORF), ORF1 protein (ORF1p) binds to RNA, ORF2 protein (ORF2p) has reverse transcriptase and endonuclease function.^[6]^ LINE-1 is a mobile DNA element that can copy and paste into new sites in the genome, it leads to gene expression changes and chromosomal instability, which can cause lesions in tissues such as tumors and nervous system.

Many causes of spontaneous abortion (SA) have been identified, such as maternal genital tract abnormalities, endocrine and immune dysfunction, sperm problems, genital tract infections, cervical insufficiency, thrombotic varices, and chromosomal abnormalities.^[7]^ About 50% to 60% of SA have karyotype abnormalities such as autosomal imbalance translocation, polyploid, X monomer, autosomal monomer, chromosome balance translocation, deletion, chimerism, inversion, overlap and others.^[8]^ During embryonic development, a single fatal gene mutation can also lead to embryonic death.^[9]^ Moreover, epigenetic abnormalities may be the cause of some early pregnancy loss.^[10]^ In recent years, the role of the placenta in embryonic development was found, which adds another layer of complexity to the abortion phenomenon.^[11]^ However, only about 50% of recurrent pregnancy loss of 3 consecutive pregnancies at least 24 weeks prior to pregnancy can determine the cause.^[12]^ Therefore, more research is needed to find the causes of abortion.

LINE-1 insertions in germ cells can transmit genetic information to the next generation via vertical transmission. We speculated that LINE-1 affects the shear and expression changes of the mRNA of genes by forming insertion mutants, which may result in abnormal embryonic development and lead to early abortion.^[13]^ There are other reports of LINE-1 and abortion. Vasil’ev et al^[14]^ examined the methylation status of LINE-1 in the early SA of aneuploid fetuses and placental tissues with normal karyotype, and found a higher LINE-1 demethylation activity in the chimested aneuploid group and the insertion of LINE-1 might lead to the chimera and produce abortion. He et al^[15]^ found that the methylation status of LINE-1 was inconsistent in the placenta of different stages and was lower in early placenta. The aim of this study is to find a new mechanism of SA: LINE-1 insertions in embryo cells lead to early SA.

## 2. Materials and methods

### 2.1. Samples

Prospective analysis of 20 early SA patients (Pregnancy ≤ 8 weeks; Normal Karyotype) and 10 induced abortion (IA) samples seen at Northwest Women and Children’s Hospital from August 2019 to December 2020. Selection criteria: All patients meet the relevant diagnostic criteria for SA in early pregnancy; with complete medical records. Exclusion criteria: with severe gynecological diseases; with malignant tumors; with hematologic diseases. Patient age was 20~48 years, with mean (28.79 ± 1.21) years. Among them, 23 were aged ≤ 36, 7 were > 36. All women and their families joined in the experiments voluntarily and signed informed consent for the experiments. The experiments were approved by Ethics Committee of Northwest Women’s and Children’s Hospital, Medical School of Xi’an JiaoTong University (No.2020-490).

### 2.2. G banding chromosome karyotype analysis

Embryonic tissue was collected under sterile conditions. The villi were separated under a dissecting microscope, it was followed by digestion with 0.25% trypsin with the addition of AmnioMAX^TM^ II media produced from Gibco Company. Primary culture was performed by straw mixing on cover slides at 1 × 10^6^ cell density, growth of the cells was recorded after 7~9 days, another 40 μg/mL of colchicine from Sigma-Aldrich company was added when the cells were cloned and covered with 80% of the cover slides, cells were extracted after 2 hours of culture. Chromosomes were observed after G dominant band production, and statistical scores were analyzed based on chromosome structure and number changes.

### 2.3. Western blot

RIPA Lysis Buffer was employed to extract total abortion tissue proteins which was quantitated by BCA Protein Quantitative Kit. After glue filling and cataphoresis, closed solution dilution of ORF1 (EMD Millipore Company 1:1000, mouse anti human) and β-actin (Weiao Company 1:2000, mouse anti human) were added to the incubation bag and incubated overnight at 4°C. The TBST membrane was washed 3 times for 5 minutes, and the horseradish peroxidase-labeled sheep anti-mouse secondary antibody (Jackson Company 1:2000) was incubated for 2 hours at room temperature. The TBST membrane was washed 5 times for 15 minutes. The membrane was reacted in chemiluminescence detection reagent (reagent A: reagent B = 1:1) for 2 minutes, the membrane was removed, the excess liquid was discarded, the PVDF membrane was wrapped with plastic wrap, X film in the dark chamber.

### 2.4. Immunohistochemistry

After dehydration, embedding, slicing, dewaxing, hydration, antigen repair by high pressure pot were carried out, inactivation of endogenous peroxidase was done by adding 1 antibody (LINE-1 ORF1, EMD Millipore Company 1:1000, mouse anti human) and horseradish peroxidase-labeled sheep anti-mouse secondary antibody (Jackson Company 1:2000). Thereafter, using hematoxylin-eosin staining, ethanol dehydration, and sealed by resin, the tissues were observed under a microscope.

### 2.5. Reverse transcription-polymerase chain reaction (RT-PCR)

Total SA and IA RNA was isolated with Trizol reagents (Invitrogen, Carlsbad, CA) as previously described.^[16]^ The RT-PCR reaction was carried out by taking 10 μg of RNA; thereafter, 2 μL Oligo dT (50 pM/μL) primer was added, diethyl pyrocarbonate water was also added to total 12 μL volume, and the mixture was incubated in 65°C water for 5 minutes. Thereafter, the following were added, 1 μL of RiboLock RNase Inhibitor, 4 μL 5X Reaction Buffer, 2 μL dNTP Mix, 1 μL RevertAid Reverse Transcriptase (total volume 20 μL) and the mixture was incubated in 42°C water for 1 hour, then cDNA template was obtained and PCR assay was carried out.

The PCR reaction system is as follows: 5 μL of 2 × SYBGEEN PCR mix, l μL each of upstream and downstream primers (10 μM, Table 1), 2.5 μL of cDNA template, with the addition of 10 μL water. The reaction parameters are 95°C denaturation for 2 minutes, 95°C for 5 seconds, 60°C for 10 seconds, with a total of 45 cycles; the last was done at 72°C for 10 minutes. After the reaction, results were observed using 8 μL PCR products, 1% agarose gel and 100 V, 30 minutes electrophoresis.

**Table 1:** RT-PCR primers.

No	Primers	Sequence(5’->3”)
1	N-51-Fwd	GAATGATTTTGACGAGCTGAGAGAA
	N-51-Rev	GTCCTCCCGTAGCTCAGAGTAATT
2	L1 5’UTR forward	ACGGAATCTCGCTGATTGCTA
	L1 5’UTR reverse	AAGCAAGCCTGGGCAATG
3	1HsL15’UTR-L-Ramos	AGCCTAACTGGGAGGCACCC
	2HsL15’UTR-R-Ramos	GATGATGGTGATGTACAGATGGG
4	ORF1-fwd	AGGAAATACAGAGAACGCCACAA
	ORF1-rev	GCTGGATATGAAATTCTGGGTTGA
5	aL1-Fw-Coufa1	GCATTACCATTCAGGACATAGGCGT
	bL1-Rv-Coufa1	GCGATTCCTCAGGGATCTAGAAC
6	c-L1-For-Menendez	TCATAAAGCAAGTCCTCAGTGACC
	d-L1-Rev-Menendez	GGGGTGGAGAGTTCTGTAGATGTC
7 (inner control)	Human B-actin F-primer	GCAGAAGGAGATCACTGCCCT
	Human B-actin R-primer	GCTGATCCACATCTGCTGGAA
8 (inner control)	Human GAPDH F-primer	GCGAGATCCCTCCAAAATCAA
	Human GAPDH R-primer	GTTCACACCCATGACGAACAT
9(inner control)	GACTBPAIR2-FOR	TTCCAGCCTTCCTTCCTG
	HACTBPAIR2-REV	AATGATCTTGATCTTCATTGTGC

Reference:^[24–26]^, RT-PCR = reverse transcription-polymerase chain reaction.

### 2.6. Statistical analysis

SPSS software version 21.0 was used for statistical analysis. The comparison of protein and mRNA levels between SA and IA, was done using the Student’s *t* test. A *P* value <.05 was considered to be statistically significant and was presented in the charts.

## 3. Results

### 3.1. LINE-1 protein expression in SA and IA

**Figure 1.**
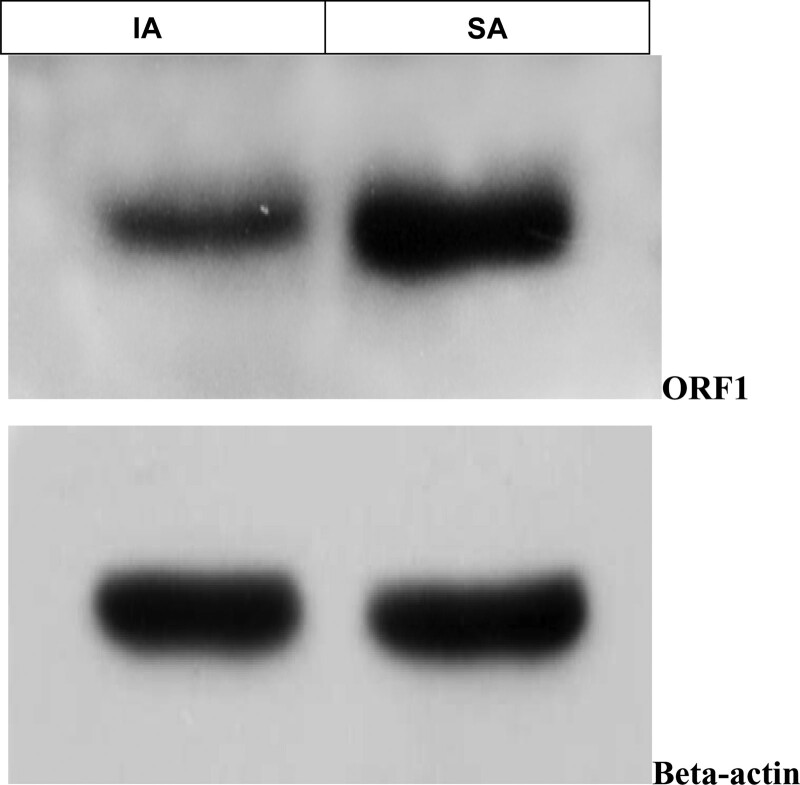
Western Blot: LINE-1 ORF1 expression in SA is much higher than in IA. LINE-1 = long interspersed nuclear element-1, ORF = open reading frame, SA = spontaneous abortion.

**Figure 2.**
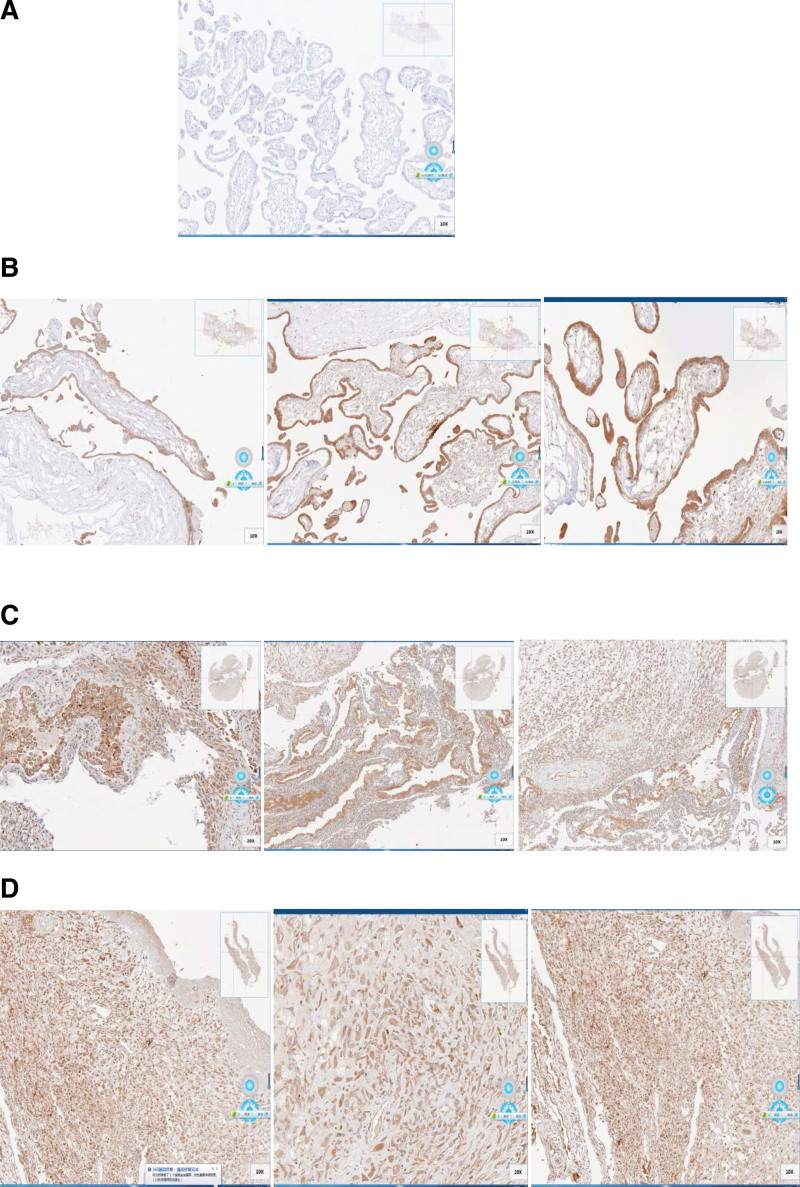
LINE-1 protein expression in SA, IA, and SA placenta. (A) Abortion microvilli negative control (HE*100); (B) IA tissue microvilli LINE-1 ORF1 expression (HE*100); (C) SA tissue LINE-1 ORF1 expression (HE*100); (D) SA placenta LINE-1 ORF1 expression (HE*200). In compared SA/IA samples, LINE-1 protein is highly expressed in SA (Mean: 59.2%) relative to IA (Mean: 28.8%), *P < .05. IA = induced abortion, LINE-1 = long interspersed nuclear element-1, ORF = open reading frame, SA = spontaneous abortion.

LINE-1 protein expression levels represent the activity of LINE-1 function. We compared the expression difference between LINE-1 protein expression in SA and IA by Western Blot (Fig. 1 and Table 2) and immunohistochemistry (IHC) (Fig. 2A–C). Both Western Blot and IHC are semi-quantitative protein detection methods, and a software was used to scan the intensity. In the Western Blot group, we compared 35 pairs of SA and IA samples, 32\35 (91%) showed that the LINE-1 protein expression in SA tissues is higher than in IA tissues. In the IHC group, we compared 30 pairs of SA and IA samples, 25\30 (83%) showed that the LINE-1 protein expression in SA tissues is higher than in IA tissues. After SPSS software calculation, LINE-1 protein is highly expressed in SA relative to IA, *P* < .05. IHC was also carried out in SA placenta tissues, and LINE-1 protein-positive expression in placenta (Fig. 2D).

**Table 2:** Western Blot intensity.

**Group**	**KD**	**IA**	**SA**
ORF1intensity	42	7148.7	22952
Beta-actinintensity	42	12000	13141
ORF1/Beta-actin		0.595725	1.746594628

The intensity of SA is 3 times higher than the intensity of IA. In compared SA/IA samples, LINE-1 protein is highly expressed in SA (Mean: 60.2%) relative to IA (Mean:30.3%), *P < .05.

IA = induced abortion, KD = kDa, SA = spontaneous abortion.

### 3.2. *LINE-1* mRNA *expression in SA and IA*

**Figure 3.**
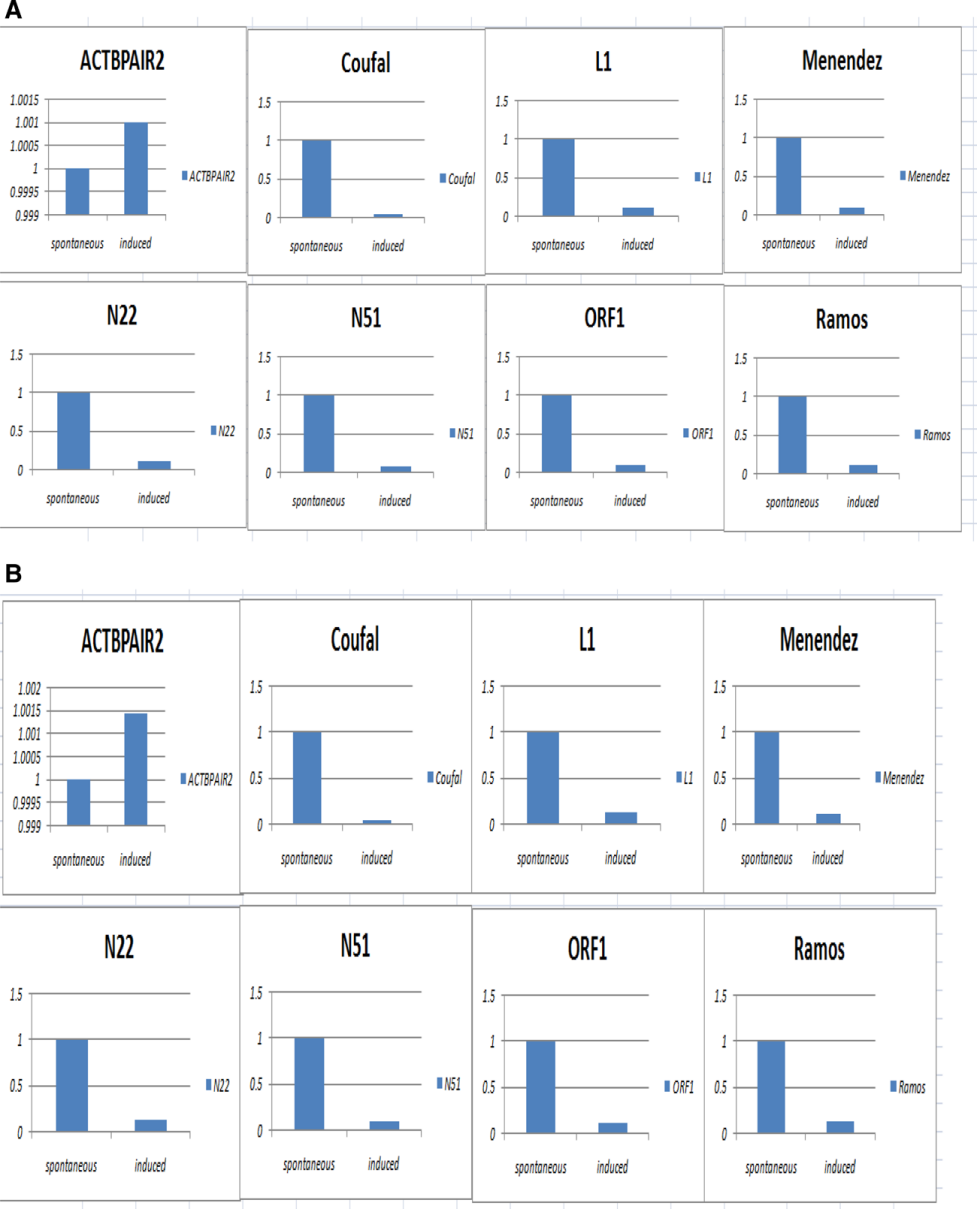
Comparison of RT-PCR results between SA and IA with different LINE-1 primers. (A) Human GAPDH as internal control; (B) Human B-actin as internal control. The 6 primers showed that the LINE-1 mRNA expression in SA is much higher than in IA. In compared SA/IA samples, LINE-1 mRNA is highly expressed in SA (Mean:64.2%) relative to IA (Mean:29.2%), *P < .05. IA = induced abortion, LINE-1 = long interspersed nuclear element-1, RT-PCR = reverse transcription-polymerase chain reaction, SA = spontaneous abortion.

**Figure 4.**
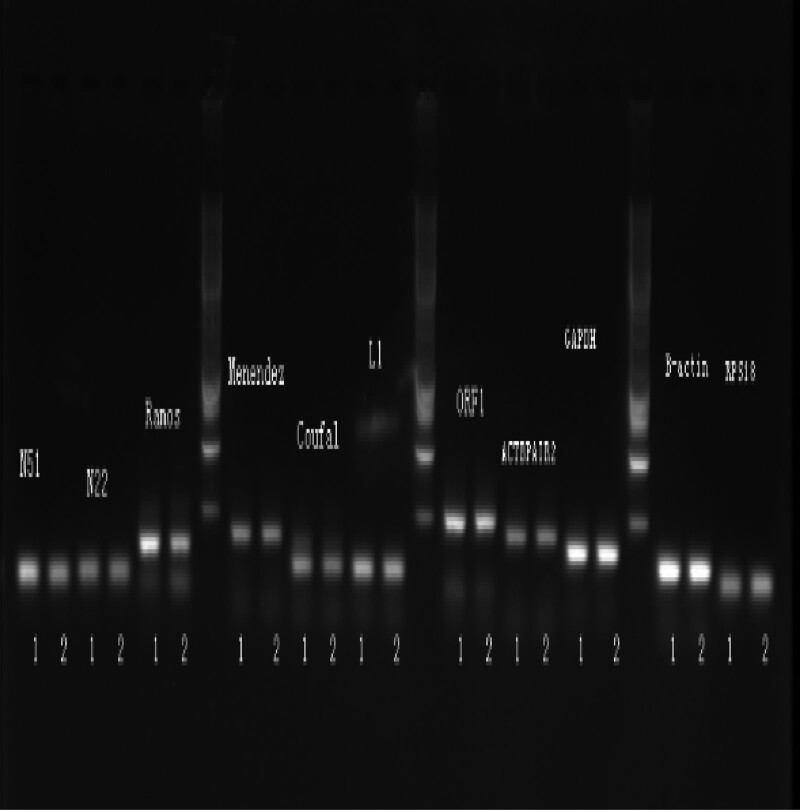
RT-PCR products of N51, N22, Menendez, Coufal, L1, ORF1, ACTBPAIR2, GAPDH, B-actin primers; 1: SA; 2: IA. IA = induced abortion, ORF = open reading frame, RT-PCR = reverse transcription-polymerase chain reaction, SA = spontaneous abortion.

To further detect the quantitative expression of LINE-1, RT-PCR was used next. We used 6 LINE-1 RT-PCR primers to represent different sites on LINE-1 mRNA. The Coufal is the most significant primer used to compare the LINE-1 expression in SA and IA (nearly 11 times higher). The other primers also showed that the LINE-1 mRNA expression in SA is much higher than in IA. GAPDH (Fig. 3A) and B-actin (Fig. 3B) were chosen as internal controls for the standard of the RT-PCR experiments. ACTBPAIR2 is another internal control. We can find that the difference between SA and IA is more obvious based on ACTBPAIR2. After SPSS software calculation, it was shown that LINE-1 mRNA is highly expressed in SA relative to IA, *P* < .05. The destruction of aborted tissue by LINE-1 depends mainly on: The number of LINE-1 insertions: More insertions are more likely to destroy chromosomal group stability and lead to abortion. LINE-1 inserted into lethal genes for embryonic development. Lethal genes are essential for normal embryonic development and are prone to miscarriage after destruction. Although LINE-1 mRNA was expressed higher in SA than IA at 6 different sites, it did not indicate that LINE-1 insertions are more frequently in SA than in IA, but it still demonstrated that LINE-1 is more active in SA than IA. RT-PCR products of different primers are also shown in Figure 4.

## 4. Discussion

SA is a common clinical problem that mainly occurs in the first 3 months of pregnancy and is mostly caused by fetal chromosomal abnormalities. Repeated abortions not only endanger women’s health, but also cause family and social problems. Presently, the rate of SA in China is about 3.6%.^[17]^ Chromosomal abnormalities account for 50% to 60% of SA, and transposon insertion is an important reason for chromosomal abnormalities.^[18,19]^

It is estimated that, more than 2 thirds of the human genome is made up of repetitive DNA and most are retrotransposons. It is a “copy and paste” mechanism where RNA intermediates are transferred to cDNA via reverse transcription, and cDNA copy is inserted into the new site of the genome. In reverse transcription, LINE-1 encoded endonuclease marks a 3’ hydroxymethyl on the target DNA chain in chromosome, to initiate the LINE-1 RNA and synthesis of cDNA at insertion sites, a process called target primed reverse transcription.^[20]^ In addition, LINE-1 is involved in the insertion of more than 10,000 processed pseudogenes and approximately 1 million short interspersed nuclear elements, including Alu and VNTR-Alu elements. When the control LINE-1 is weakened, LINE-1 can have a huge impact on other genes. The first is the effect on its adjacent genes: LINE-1 insertion brings new shear sites, PolyA addition signals, promoters, and corresponding transcription factor binding sites to the surrounding genes, all these factors affect or even reconstruct the expression of surrounding genes. Moreover, LINE-1 sometimes brings part of the sequences adjacent to its 3’or 5’ end into a new chromosome position during the transposition process, thus causing gene rearrangement or producing new genes. Except for the influence of transposon itself on adjacent genes, the LINE-1 scattered in the genome will increase the probability of homologous recombination between different genes, and lead to the duplication or deletion of large fragments of genes. For example, the insertion of retrotransposons in chromosomes can damage chromosome structure,^[21,22]^ which can cause the formation of chimerism, deletion, repetition, inversion, translocation and aneuploidy, and may lead to abnormal fetus development and abortion.

Although Western Blot was semi-quantitative, intensity of LINE-1 in SA was 3 times higher than in IA. We used 6 LINE-1 RT-PCR primers to represent different sites on LINE-1 mRNA and found significant difference between SA and IA. From the IHC results, we can also find differences in LINE-1 expression. Our results demonstrated that LINE-1 expression in SA is significantly higher than in IA both from protein and mRNA levels, indicating that LINE-1 is more active in SA and may play a role in abortion. Although we cannot show that LINE-1 has many insertions in embryonic cells, we still need sequencing analysis to find if there are substantial insertions. Moreover, we also found LINE-1 in the IHC of the placenta, and the role of LINE-1 in causing abortion still remains to be investigated.

Human cells have evolved a variety of defense mechanisms to limit retrotransposenic mutations, but these defense mechanisms may not be perfect in early human embryonic cells. LINE-1s in germ cells or early embryos may lead to over-mutations of the genome, or inflammatory response and apoptosis as a result of increased expression of nucleic acids and proteins from LINE-1 sources, thus impairing the normal development of the embryos. We hope to find a new cause of early human abortion, LINE-1 insertion may be a key point of the known network that causes abortion such as chromosomal structural changes, aneuploidy changes and mutations in abortion-related genes, and form an inter-connected network to deepen the understanding of the mechanism of abortion and to provide a new therapeutic strategy for the control of early recurrent abortion. If the check on abortion tissues shows that some unexplained recurrent abortions were caused by increased LINE-1 activity, treatment advice can be proposed. Injecting low doses of reverse transcription inhibitors to these patients in early pregnancy can decrease the incidence of the future abortion.

Therefore, we assumed that the insertion of retrotransposon LINE-1 is a new mechanism leading to abortion, and it is important to explore its molecular mechanism. The verification of this hypothesis can find a new mechanism of abortion to guide and prevent the clinical diagnosis and treatment of abortion patients. We still need to investigate more abortion samples to prove this hypothesis. If it is confirmed that retrotransposon activity has a large impact on fetal damage in some patients, administering a low dose of retrotransposition inhibitors may reduce the incidence of recurrent abortion in the future. Moreover, in cell culture experiments, L1 reverse transcription was strongly inhibited by a nucleoside reverse transcriptase inhibitor.^[23]^

## Author contributions

**Data curation:** Liping Zhang.

**Formal analysis:** Ting Jia.

**Methodology:** Chao Lou, Hanzhi Wu, Wei Li, Ting Jia.

**Resources:** Hanzhi Wu.

**Visualization:** Liping Zhang.

**Writing – original draft:** Chao Lou, Xing Liu.

**Writing – review & editing:** Rong Qiang, Xing Liu.
